# Dual infection with *Acinetobacter baumannii* and *Klebsiella pneumoniae* in a patient with multiple comorbidities – case presentation

**DOI:** 10.1186/1471-2334-14-S7-P26

**Published:** 2014-10-15

**Authors:** Oana Săndulescu, Monica Andreea Stoica, Ioana Berciu, Anca Streinu-Cercel, Liliana Lucia Preoțescu, Adrian Streinu-Cercel

**Affiliations:** 1 Carol Davila University of Medicine and Pharmacy, Bucharest, Romania; 2 National Institute for Infectious Diseases "Prof. Dr. Matei Balş", Bucharest, Romania

## Background

Bacterial coinfection is a rarely described phenomenon. We present a case of dual infection in a patient with multiple comorbidities and advanced immune-suppression.

## Case report

A 35 year-old male patient presented to our clinic in May 2014 for progressive malaise, low-grade fever and nausea.

His medical history revealed chronic glomerulonephritis and renal failure with hemodialysis from 2005 to 2010; kidney transplant in 2010, with transplant rejection and positive CMV-IgM in March 2014. He also presented arterial hypertension, ischemic heart disease and left ventricular hypertrophy since 2010, multiple episodes of sepsis and pneumonia with *Klebsiella* spp. through digestive microbial translocation (colonic ulcerations), and a double aortocoronary bypass in March 2014. The thoracotomy incision had healed almost completely, but the right calf incision presented signs of infection.

His concomitant therapy included anti-hypertensive agents, antiplatelet therapy, ganciclovir, immune-suppression therapy with mycophenolic acid, and prednisone (10 mg/day).

On admission, the clinical exam was normal, except for bilateral lower limb edema and inflammation of the right calf incision area, with multiple patches of exposed soft tissue and suppuration. Biologically, he presented pancytopenia (WBC 2,100 cells/µL, hemoglobin 6.8 g/dL, thrombocytes 137,000 cells/µL), nitrogen retention syndrome (urea 147.4 mg/dL, creatinine 4.4 mg/dL). The patient’s reactivity was quite low given the concomitant immune-suppressive treatment, with ESR 38 mm/1h, fibrinogen 351 mg/dL, and CRP 10 mg/L.

Urine cultures, repeated blood cultures and procalcitonin were negative, but the smear from the right calf incision wound identified inflammatory cells and Gram-negative coccobacilli, and CLED cultures grew smooth, yellow, lactose-fermenting colonies. Microscan (Siemens, Munich, Germany) identified carbapenemase-producing *Klebsiella pneumoniae* (KPC) and the strain was subcultured and grew a smooth, grey, non-lactose-fermenting colony, identified on VITEK (bioMérieux, Paris, France) as *Acinetobacter baumannii*. Both strains were resistant to all tested drugs except for colistin and tigecycline. As both strains initially grew in a single isolated culture, with homogenous morphology, it took repeated cultures to separate the two strains. The patient’s evolution was favorable under treatment with tigecycline and local instillations of colistin.

## Conclusion

We have presented a case of coinfection with two extremely resistant Gram-negative strains in a patient with multiple comorbidities and limited treatment options. Interestingly, both strains grew entwined in a colony that presented a single morphology not suggestive for coinfection. Bacterial identification techniques allowed an etiologic diagnosis and targeted antimicrobial therapy.

